# Designing and implementing SMILE: An AI-driven platform for enhancing clinical decision-making in mental health and neurodivergence management

**DOI:** 10.1016/j.csbj.2025.02.022

**Published:** 2025-02-22

**Authors:** Antonio Pesqueira, Maria Jose Sousa, Ruben Pereira, Mark Schwendinger

**Affiliations:** a ISCTE-IUL Av. das Forças Armadas, Lisboa 1649-026, Portugal; b University of Maryland, College Park, MD 20742, United States

**Keywords:** Mental health, Neurodivergence, Dynamic capabilities, Cognitive behavioral therapy, Artificial intelligence

## Abstract

Rising levels of anxiety, depression, and burnout among healthcare professionals (HCPs) underscore the urgent need for technology-driven interventions that optimize both clinical decision-making and workforce well-being. This innovation report introduces the Support, Management, Individual, Learning Enablement (SMILE) platform, designed to integrate advanced AI-driven decision support, federated learning for data privacy, and cognitive behavioral therapy (CBT) modules into a single, adaptive solution. A mixed-methods pilot evaluation involved focus groups, structured surveys, and real-world usability tests to capture changes in stress levels, user satisfaction, and perceived value. Quantitative analyses revealed significant reductions in reported stress and support times, alongside notable gains in satisfaction and perceived resource value. Qualitatively, participants praised SMILE’s accessible interface, enhanced peer support, and real-time therapeutic interventions. These findings confirm the feasibility and utility of a holistic, Artificial Intelligence (AI) supported framework for improving mental health outcomes in high-stress clinical environments. Theoretically, SMILE contributes to emerging evidence on integrated AI platforms, while it offers an ethically sound and user-friendly blueprint for improving patient care and staff well-being.

## Graphical Abstract

**Figure:**
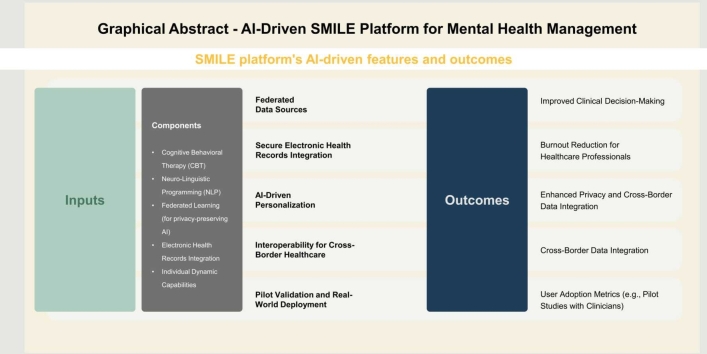

## 1 Introduction

Healthcare systems worldwide face escalating challenges in mental health and central nervous system care. Growing rates of anxiety, depression, and burnout among healthcare professionals (HCPs) not only threaten patient outcomes but also compromise workforce stability. Addressing this crisis demands more than incremental improvement; it necessitates comprehensive, technology-driven interventions that can support both patient care and the mental well-being of clinicians. In particular, artificial intelligence (AI) offers transformative opportunities to empower data-driven clinical decision-making, personalize treatment plans, and facilitate continuous training for HCPs [22]. However, deploying such AI-based solutions also entails addressing stringent data protection requirements, fostering clinician trust data protection, and ensuring practical integration with existing workflows. Solving these interconnected problems in mental health is essential for two key reasons (Bhattamisraet al.,2023). First, any meaningful advancement in mental health care must also address HCP resilience, as providers grappling with burnout often deliver suboptimal care. Second, bridging technological innovations with ethical and regulatory compliance remains critical in modernizing health systems. A robust AI-enabled solution can bring about efficiencies—such as shortened administrative times and enhanced diagnostic support—thereby freeing clinicians to devote more attention to individualized care, patient engagement, and evidence-based therapeutic practice [3], [7].

Recent research has identified the potential of AI to support clinical workflows, reduce errors, and facilitate mental health interventions. However, unresolved issues persist. Clinical learning and continuous medical support systems lack provisions to manage clinicians' stress levels. Solutions tailored for provider well-being often overlook data-sharing risks and confidentiality concerns [8]. These solutions also often fail to ensure full protection and privacy, which is crucial in preventing stigma or negative bias from peers and employers. Recent literature on individual dynamic capabilities (IDC) has emphasized the necessity of organizational and personal adaptability in integrating modern technologies into complex healthcare environments [5]. While AI can facilitate this adaptability, it is crucial to employ it responsibly to address mental health concerns, both for patients and clinicians. Furthermore, while cognitive behavioral therapy (CBT) and neuro-linguistic programming (NLP) interventions have demonstrated efficacy in the mitigation of stress, the majority of existing platforms treat these therapies as discrete components rather than integrating them with clinical data or organizational performance metrics [30].

The Support, Management, Individual, Learning Enablement (SMILE) platform integrates AI-driven training and learning support, blockchain-based identity management and personal identification data protection, and targeted CBT modules into one cohesive framework. SMILEs’ primary goals are: (1) to reduce clinical workload and stress through adaptive, data-driven insights; (2) to preserve robust data privacy by leveraging federated learning and decentralized ledgers; and (3) to reinforce IDC among HCPs, thereby improving resilience and the adoption of innovative healthcare technologies. Supplementary objectives include evaluating the impact of AI-based mental health modules on clinician satisfaction, exploring cross-border compatibility with regulations such as the General Data Protection Regulation (GDPR), and assessing the feasibility of scalable deployment in diverse settings. The present research problem emerges from a clear gap in integrated solutions: while AI tools for mental health abound, few systems effectively combine advanced analytics, secure data governance, and direct therapeutic interventions aimed at clinicians themselves. SMILE’s structured design positions it to address this gap by embedding CBT/NLP into daily workflows and using federated learning to maintain confidentiality. The study employed a phased methodology encompassing controlled simulations, pilot deployments in neurology and psychiatry departments, and iterative feedback loops. Data sources included baseline and post-implementation stress scores, user satisfaction surveys, value assessments, and focus group interviews. Two parallel avenues of analysis—quantitative metrics for efficacy and qualitative insights for usability—enabled a nuanced understanding of the platform’s real-world viability.

From a theoretical standpoint, this innovation report extends evidence on the convergence of AI and IDC frameworks within healthcare contexts. It responds to pressing concerns about burnout and limited interoperability by illustrating how carefully designed AI modules, combined with robust blockchain-based data tracking, can protect privacy, and streamline collaboration. In turn, SMILE’s findings open new avenues for refining integrative solutions that cater to clinicians’ emotional well-being and foster organizational agility. Finally, the expected practical contributions of SMILE include concrete guidelines for designing ethically aligned AI solutions—encompassing compliance with GDPR and the EU AI Act—and operational frameworks that optimize clinician engagement and mental health support. By bridging IDCs with data privacy safeguards, SMILE offers a model for reducing stress, enhancing decision-making, and reinforcing collective resilience. Such an approach, if widely adopted, could reshape healthcare organizations’ strategies, promoting sustainable, inclusive, and future-ready systems.

## 2 Relevant literature

### 2.1 Data security, regulations, and standards

The incorporation of AI-driven solutions into mental healthcare necessitates stringent attention to data security, regulatory compliance, and standardized processes. Over the past decade, scholarly discourse has increasingly centered on the multifaceted challenges inherent in safeguarding patient information while advancing clinical decision support systems (CDSS). A foundational theoretical perspective for understanding the adoption of complex technological systems in healthcare is rooted in dynamic capabilities (DC). DC theory posits that organizations possess the capacity to sense, seize, and reconfigure resources to maintain competitiveness in rapidly shifting environments [10]. Within the healthcare sector, this lens has been adapted to address the organizational reorientation required for adopting AI-driven platforms [13]. Central to DC theory are processes that facilitate continuous learning, strategic decision-making, and technological innovation to support evolving clinical needs. Relatedly, at the individual level, IDC underscores HCP's adaptability, encompassing cognitive flexibility, emotional resilience, and proficiency in recent technologies. By emphasizing these interrelated capabilities, contemporary research highlights the importance of both organizational and personal readiness in effectively integrating AI solutions [19], [27].

Building on these theoretical positions, scholars have surveyed the proliferation of AI in healthcare, offering methodological insights into data security and regulatory compliance. Early works focused on conventional data management frameworks, highlighting encryption and secure data transmission as preliminary strategies for preventing breaches. More recent literature has expanded its scope by examining advanced approaches such as blockchain technology, federated learning, and zero-knowledge proofs [25]. Federated learning, for instance, enables distributed model training by ensuring that patient data remains on local devices or servers, thus minimizing potential privacy violations while fostering collaborative model development across multiple institutions. Blockchain systems introduce immutable, transparent ledgers that facilitate reliable identity management and consent tracking, effectively reinforcing trust among stakeholders [27], [37]

Despite these methodological developments, distinct debates persist in literature. A principal contention arises around the trade-off between innovation and regulation, particularly within highly regulated sectors such as mental healthcare. On one side of the debate, advocates argue that stringent regulatory frameworks—such as the GDPR and the emerging EU AI Act—are essential for maintaining patient privacy, ensuring informed consent, and upholding ethical AI practices [10]. Conversely, critics caution that excessively rigid regulations may stifle innovation and hamper the development of AI-driven CDSS capable of addressing urgent clinical needs, especially in mental health contexts. These perspectives contend that prolonged regulatory approval cycles and complex data handling requirements slow the adaptation of advanced technologies and limit HCPs’ ability to respond rapidly to patient crises [16].

A second debate focuses on the scope and efficacy of existing standards. Although standards such as Health Level 7 (HL7) Fast Healthcare Interoperability Resources (FHIR), the 10th revision of the International Classification of Diseases (ICD-10), and the International Organization for Standardization (ISO)/International Electrotechnical Commission (IEC) 27001 provide valuable guidance for data exchange, disease classification, and information security, some researchers question the extent to which these frameworks truly foster interoperability. Interoperability challenges become especially pronounced in mental healthcare, where data results from diverse clinical settings, including psychiatry, psychology, social work, and community-based services [3], [9]. Furthermore, the semantic depth required for capturing nuanced mental health presentations far surpasses conventional diagnostic categories in ICD-10, calling for more granular and adaptable classification systems (Ullahet al., 2023). Critics thus contend that while universal standards are critical for ensuring a consistent baseline of quality, they do not necessarily accommodate emerging data types, personalized interventions, and the evolving demands of AI-driven therapies [29].

A counterargument to these concerns is that the ongoing evolution of standards, spurred by initiatives such as the European Patient Summary (EPS) and updated versions of ISO/IEC 27001, demonstrates a concerted effort to harmonize healthcare IT infrastructures. Proponents of this position emphasize that recent revisions in GDPR compliance guidelines and the push for more robust identity and access management protocols suggest a dynamic regulatory environment designed to adapt to technological change [21]. These measures, they argue, enable healthcare institutions to fine-tune their security strategies, develop advanced consent management mechanisms, and maintain trust among patients and professionals alike. Nevertheless, even supporters of stringent regulations acknowledge the pressing need for flexible frameworks that balance security obligations with the urgency of delivering immediate, effective mental health interventions (Tariqet al., 2022).

Within this contested landscape, a clear research gap emerges. Although scholars widely recognize the importance of robust data security and regulatory compliance, few existing frameworks focus explicitly on mental health-centric CDSS solutions that integrate adaptive therapeutic modules while maintaining stringent privacy requirements [17]. The conventional one-size-fits-all approach often fails to consider the complexities of mental health data, which may include psychotherapy notes, sensitive behavioral assessments, and continuous monitoring through wearable or home-based devices [1]. Moreover, AI-driven tools offer little insight into how HCPs can effectively develop their capabilities, particularly IDC, to accommodate evolving regulatory and interoperability mandates. This gap highlights an unmet need for novel architectures that marry advanced technical strategies with user-centric design and professional development initiatives [14].

By emphasizing European regulations, such as the GDPR, the EU AI Act, and supporting standards like HL7 FHIR, the International Classification of Health Interventions (ICHI), and the European Committee for Standardization CEN/ISO 13606, various solutions have sought to position themselves as comprehensive solutions [6], [9]. These solutions aim to comply with prevailing legal mandates and enhance the quality, scalability, and personalization of mental health interventions [2]. Therefore, the existing literature underscores the critical role of data security and regulatory compliance in the development of AI-driven mental health systems, yet significant gaps persist regarding the integration of flexible therapeutic modules, interoperability within diverse clinical contexts, and the cultivation of dynamic capabilities among HCPs. In addressing these limitations, the novel can offer the potential to harmonize innovative AI methodologies with robust security standards, thereby ensuring both the ethical integrity and the clinical utility of emerging CDSS platforms in mental health and neurodivergence management [35].

### 2.2 IDC, CBT, and NLP in Mental Health and CNS Disorders

Achieving seamless data exchange and delivering effective interventions for mental health and central nervous system (CNS) disorders requires an intricate balance between robust interoperability frameworks and evidence-based therapeutic methodologies. Recent literature highlights the importance of open systems that enable the fluid sharing of clinical insights, patient records, and decision-support tools across diverse healthcare environments[18], [23]. Despite this consensus on the transformative potential of interoperability, the reality characterized by siloed data repositories, regulatory mismatches, and varying levels of technological infrastructure. At the same time, the role of IDC among HCPs has become more pronounced, particularly as AI reshapes diagnostic and therapeutic processes. While DC theory explains how organizations adapt to rapidly shifting environments, IDC emphasizes the need for HCPs to cultivate cognitive flexibility, emotional resilience, and technological fluency to integrate AI-driven systems effectively into their daily workflows [12].

One of the principal debates in this domain revolves around balancing the urgency of immediate data access with the imperative of patient privacy. Advocates argue that cross-institutional and cross-border interoperability is essential for enhancing mental health and CNS care, as it allows practitioners to build upon shared patient histories and expedite treatment decisions [20]. Conversely, skeptics caution that unregulated data exchange risks exposing sensitive patient information to breaches, thus necessitating stringent adherence to regulations such as the GDPR and the emerging EU AI Act. Scholars on both sides of the debate acknowledge that emerging solutions, such as federated learning and blockchain-based identity management, offer considerable promises for reconciling these competing interests. Nonetheless, the literature remains sparse on large-scale, empirical validations of these technologies, specifically within mental health contexts—a gap that signals the need for platforms like SMILE to demonstrate how advanced encryption, immutable ledgers, and distributed AI model training can simultaneously foster data fluidity and protect patient confidentiality [15].

Another key challenge to effective interoperability involves the uneven application of established standards for electronic health records and data exchange. Frameworks such as HL7 FHIR and CEN/ISO 13606, combined with initiatives like the European Patient Summary (EPS), have formalized protocols for transferring clinical information consistently (Koubaet al., 2023). However, certain regions with limited digital resources struggle to implement these standards, leading to fragmented systems that impede the seamless flow of information [24]. Furthermore, cultural nuances and divergent regulatory policies across countries can undermine even the most well-designed interoperability solutions, particularly in mental health settings where data types may extend beyond conventional lab results or imaging to include sensitive notes, psychological assessments, and social determinants of health. While adaptive data mapping technologies and multilingual interfaces offer partial remedies, there is an evident gap in research examining how these tools can be scaled and contextualized for different healthcare ecosystems [4]. Proponents of broader international collaboration assert that engaging global bodies and regional authorities in co-developing data standards could pave the way toward truly border-transcending mental healthcare. Detractors, however, suggest that the logistical demands of orchestrating multi-jurisdictional data flows may be unrealistic without substantial structural reforms [6], [32], [33].

Within this landscape of evolving technical and regulatory demands, CBT and NLP have attracted growing attention as foundational psychotherapeutic approaches that could be integrated into next-generation CDSS. CBT’s theoretical core, centered on identifying and modifying maladaptive thoughts and behaviors, has established it as a gold-standard intervention for an array of mental health conditions. NLP, though less uniformly embraced, explores how linguistic patterns and subconscious programming can be reshaped to alleviate anxiety and enhance resilience [34], [36]. Both methods share an emphasis on building cognitive flexibility, a skill that resonates with the concept of IDC by preparing both patients and HCPs to adapt proactively to shifting clinical landscapes. Coupling these therapeutic frameworks with AI has enabled personalized assessments, immediate feedback, and data-driven insights into patient progress. For instance, AI modules can parse linguistic markers in real-time to detect cognitive distortions, prompting timely interventions that may prevent exacerbations of mental health symptoms (Gebhardtet al.,2022).

Yet, despite the growing evidence that AI-powered applications can extend the reach of CBT and NLP, the literature reveals a dearth of cohesive models that merge these interventions with robust interoperability protocols. Existing studies often concentrate on either the psychotherapeutic efficacy of CBT and NLP or the technical dimensions of AI-based systems without fully exploring how these elements might coalesce in large-scale deployments. This fragmentation is particularly notable in mental healthcare, where cross-institutional collaboration and secure data exchange are integral to developing holistic treatment plans [26]. Researchers have posited that advanced CDSS built on federated learning architectures and blockchain verification could unify these disparate research threads, offering real-time capabilities for clinicians to track patient progress, collaborate with peers, and update treatment protocols dynamically. However, limited empirical analysis of whether such systems can effectively manage the delicate interplay of culture-specific therapeutic approaches, regulatory variations, and data privacy stipulations across multiple regions [31].

A parallel research gap concerns the capacity of HCPs to adapt to these rapidly changing environments. While institutional buy-in and funding are critical, IDC, encompassing the willingness to embrace technological innovations, resilience in the face of evolving regulations, and the acquisition of new clinical and analytical competencies—are essential for long-term sustainability [28]. Critics argue that in the absence of well-structured educational programs, HCPs may feel overwhelmed by the influx of innovative technologies and data governance requirements, potentially undermining the adoption and efficacy of AI-driven interventions. In response, proponents highlight simulation-based training, interactive peer learning, and structured competency frameworks as strategies to enhance IDC among clinicians, thereby bolstering the clinical impact of interoperable CDSS. Nonetheless, there remains a paucity of research on designing and implementing these programs in tandem with platform-level interoperability solutions, suggesting that the literature has yet to fully examine how professional development initiatives align with advanced technological infrastructures [11].

## 3 Project description

The SMILE platform was conceived to address the intensifying challenges that HCPs face in high-pressure environments. Anxiety, depression, and burnout now rank among the leading occupational hazards affecting HCPs, compromising both patient outcomes and the professionals’ well-being. By merging advanced technologies with evidence-based interventions, SMILE aspires to provide a holistic system that is readily adaptable to various clinical settings. Its focus on IDC and CBT aims to foster resilience, enhance clinical decision-making, and reduce daily stress levels among clinicians.

At its core, SMILE integrates three key components into an existing point-of-care ecosystem. First, the platform offers structured educational resources aligned with continuing medical education or learning management system frameworks, ensuring that HCPs remain current with evolving clinical guidelines and research developments. Whether these resources involve step-by-step tutorials on implementing new procedures or interactive sessions to master CBT fundamentals, they are continually updated and peer-reviewed to reflect the latest evidence-based practices. Second, SMILE incorporates AI-driven monitoring and personalized interventions, including CBT modules and NLP tools—that aim to reduce clinician stress and enhance patient care. By assessing user inputs, such as mood ratings or time spent on high-pressure tasks, SMILE can proactively suggest targeted strategies such as mindfulness breaks, on-demand training, or real-time peer consultation. Third, the platform employs secure data management and identity solutions, using blockchain-based consent mechanisms to protect sensitive information, address privacy concerns, and comply with regulatory standards like the GDPR. A defining feature of SMILE is its emphasis on IDC. Drawing on strategic management principles, IDC highlights the ability of individuals and organizations to adapt to a constantly shifting healthcare landscape.

Within SMILE, this takes shape through interactive training modules and guided simulations that help HCPs cultivate emotional resilience, cognitive flexibility, and technological proficiency. For instance, a primary care physician might complete a short CBT-based exercise to learn adaptive coping strategies for handling difficult patient interactions. Meanwhile, a nurse manager working under high-pressure circumstances may explore advanced scheduling algorithms integrated with AI, refining her capacity to delegate tasks and minimize workflow bottlenecks. By embedding these learning elements into daily routines, the platform fosters a culture of continuous improvement and professional education.

Another crucial aspect is SMILE’s capacity to integrate with other existing decision support systems or already operating healthcare systems for easy access. Rather than replacing the technology infrastructure already in place, SMILE augments it with value-added modules for mental health support professional development and systems interoperability. An HCP accessing a standard system can, for example, access SMILE’s AI analytics panel for stress self-assessments or quick references to specialized CBT content. This ensures minimal disruption to routine clinical tasks while opening a new avenue for real-time mental health resources. Additionally, clinicians can engage with the platform’s peer support network AI conversational channel, where encrypted communication channels facilitate private interactions and collaborative problem-solving—critical for mitigating feelings of isolation or burnout.

From an implementation standpoint, SMILE employs a phased deployment strategy. The system’s scalability and adaptability rank high among its design principles. SMILE offers multilingual support and culturally sensitive therapeutic tools, broadening its applicability in regions with diverse population demographics and healthcare priorities. Moreover, the platform’s architecture accommodates lower-bandwidth or offline scenarios, potentially expanding mental health resources into clinics with intermittent internet connectivity. By adopting federated learning, SMILE trains AI models locally on each site’s data, avoiding the centralization of sensitive clinical information while enabling the distribution of model improvements across multiple facilities. These safeguards resonate with the platform’s commitment to data ethics and secure data-sharing practices.

**Fig. 1:**
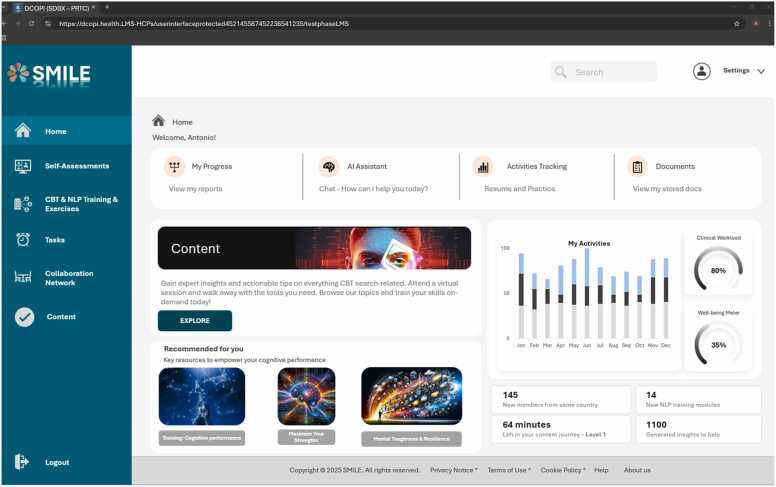
User interface – home page.

**Fig. 2:**
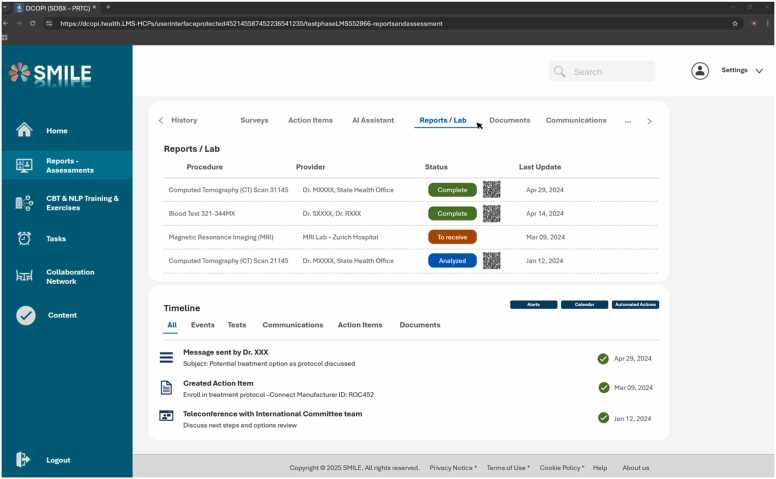
User interface – reports/assessment.

**Fig. 3:**
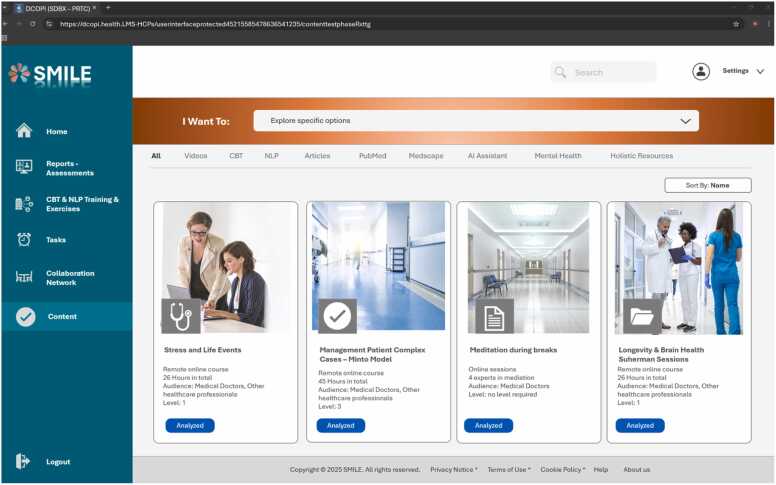
User interface – content.

The following screenshots – Fig. 1, Fig. 2, Fig. 3 - illustrate how the platform’s user interface integrates clinical workflows and personal well-being tools. Each page underscores the seamless coexistence of AI-driven recommendations, CBT/NLP training modules, and collaboration features within an approachable dashboard. In particular, the first figure presents a central hub that combines real-time activity tracking and recommended resources, highlighting SMILE’s orientation toward both clinical and self-care objectives. The second figure delves into how reports and lab data are accessed and updated, emphasizing HCPs' ability also to manage their healthcare records through secured and protected data protocols. Lastly, Fig. 3 reveals the structured content repository for exploring courses, articles, and supportive materials tailored to different professional levels.

The SMILE platform provides an integrated framework that combines clinical decision support, professional development, and mental health intervention within a single, secure system. Its emphasis on IDC assists HCPs in developing self-awareness, strategic thinking, and adaptability—characteristics essential for performing effectively in high-pressure environments. In parallel, its training modules, AI-driven monitoring tools, peer support features, and blockchain-based privacy measures collectively address the complexities of contemporary clinical practice (Table 1).

**Table 1:** SMILE project’s key elements, descriptions, and benefits.

**Element**	**Description**	**Key Benefits**
AI-Driven Decision Support	Integrates algorithms and federated learning techniques into existing CDSS, offering real-time insights into patient conditions.	Reduces clinical workload, enhances accuracy, and safeguards data privacy through decentralized model training.
CBT & NLP Modules	Provides structured CBT exercises and NLP prompts for HCP well-being.	Alleviates stress and burnout by delivering evidence-based mental health interventions targeted at clinicians’ self-care needs.
IDC	Emphasizes personal adaptability and resilience, guiding HCPs to develop cognitive flexibility and advanced problem-solving skills.	Fosters a more agile workforce prepared to manage complex and shifting clinical demands.
Blockchain-Based Consent	Uses permissioned ledgers to track data access events and authenticate user identities.	Ensures secure, tamper-proof auditing and compliance with data protection regulations such as GDPR.
Peer Support Network	Establishes secure channels for HCPs to connect and share experiences in a confidential setting.	Mitigates isolation promotes collaboration, and supports collective knowledge-sharing among team members.
LMS Integration	Embeds SMILE within a learning management system or CME platform for continuous professional development.	Keeps clinicians updated on the latest guidelines and research, reducing variability in care across different teams.
Adaptive Deployment	Offers offline-ready features and multilingual functionalities, adaptable to varying resource constraints and cultural contexts.	Expands reach to underserved regions, ensures user inclusivity, and improves accessibility for diverse clinical environments.
8. Phased Implementation and Scaling	Proceeds through pilot testing, iterative refinements, and broader expansion guided by real-world feedback loops.	Facilitates steady improvement, aligns platform features with emerging needs, and sustains meaningful long-term adoption in healthcare systems.

The project was designed as a phased intervention that could be rigorously assessed at each milestone through a combination of quantitative and qualitative methods. A six-month pilot study served as the initial demonstration phase, centered primarily on a hospital system that had shown openness to integrating AI tools. This pilot phase included iterative testing of SMILE with an existing CDSS and then with a learning and continuous medical education platform, focusing on usability, data accuracy, and responsiveness to HCP needs. Detailed records of software usage, user feedback, and decision-making patterns were collected to evaluate how effectively the system influenced both clinical outcomes and HCP well-being. Subsequently, the project expanded to more diverse healthcare settings with practitioners who had varying levels of technological proficiency, thereby capturing a broader representation of real-world adoption challenges, and learning curves. Even though the SMILE initiative has thus far been implemented solely in pilot stages within outpatient clinics, hospital neurology units, and community mental health centers, there remains a continued review process for its transition from a prototype phase to a more extensive implementation scale.

Data collection and procedure documentation were integral to assessing the platform’s efficacy and compliance. The Computerized System Validation (CSV) lifecycle provided the structural framework for verifying and documenting SMILE’s functionality, beginning with a well-defined validation plan that outlined the scope, risk assessment, and deliverables. The installation qualification phase confirmed that SMILE’s hardware and software could be seamlessly installed within existing IT infrastructures, ensuring minimal disruption to hospital networks. Also, operational qualification procedures then scrutinized the system’s core functions, including its ability to generate evidence-based mental health recommendations and safeguard sensitive data under varying workloads. Additionally, performance qualification assessed SMILE’s reliability in live clinical scenarios, including the capability of its AI-driven modules to adapt recommendations according to patient progress and HCP feedback. Each stage was documented in a requirements traceability matrix, ensuring that each specified function such as encryption protocols and interoperability with electronic medical records—was validated against the original system requirements.

The project’s outcomes were conceived in both clinical and organizational terms. Clinically, the platform aimed to refine diagnostic accuracy and therapeutic interventions for patients by integrating genomics data, treatment histories, and real-time assessments. This was measured through metrics such as error rates in diagnoses, response times to urgent mental health needs, and HCP adherence to recommended treatment pathways. Organizationally, the project’s success was judged by reduced incidence of clinician burnout, improved user satisfaction scores, and enhanced confidence in AI-led decision-making processes. In addition to these performance metrics, security, and regulatory compliance functioned as another key outcome category, reflected in successful audits of SMILE’s data protection measures and alignment with different technical guidelines. Collectively, these parameters were monitored through a combination of usage analytics, standardized questionnaires, focus group interviews, and formal data security audits conducted at each phase of the CSV lifecycle.

Analysis of the data followed a mixed-methods framework. Quantitative metrics, such as reductions in diagnostic errors or improvements in care continuity, were subjected to statistical tests comparing baseline performance against measurements captured during and after the SMILE implementation. The qualitative analysis included thematic reviews of interview transcripts and free-text survey responses, providing deeper insights into how participants adapted to the platform’s new workflow patterns, perceived the relevance of AI-driven mental health support, and addressed privacy concerns. By integrating these two analytic approaches, the SMILE team aimed to construct a comprehensive understanding of both the platform’s clinical efficacy and the organizational dynamics that either facilitated or hindered its acceptance.

Ultimately, the SMILE project was devised to offer a secure, regulatory-compliant, and user-focused system that elevates the standard of care for mental health and CNS disorders while simultaneously supporting HCPs’ well-being. Through federated learning methodologies, clinicians gained access to aggregated data insights that remained privacy-preserving, allowing them to tailor treatment plans with greater precision. The incorporation of blockchain-based consent management served as an additional layer of trust, although robust evidence of blockchain’s effectiveness at scale continues to require ongoing evaluation. Moreover, SMILE’s alignment with existing guidelines—spanning HL7 FHIR, GDPR, and the EU AI Act—demonstrated its potential to operate effectively within international data-sharing ecosystems. This emphasis on regulatory compliance was especially crucial given the substantial legal and ethical responsibilities that come with cross-border health data exchange.

### 3.1 Project phasing

The project was structured into multiple phases to ensure a user-centric, iterative, and rigorous approach, drawing on design thinking, Agile, Scrum, and Six Sigma principles. Table 2, Table 3, Table 4 -, Table 5 summarize the core activities, methods, and key outcomes for each phase, reflecting an evolving project strategy designed to integrate clinical workflows, regulatory imperatives, and mental health–specific interventions.

**Table 2:** Phase 1, empathize and define (Conceptualization).

**Step**	**Description**	**Methods**	**Key Outcomes**
Stakeholder Research	Conduct in-depth interviews, surveys, and focus groups with clinicians and policymakers.	Design Thinking Empathy Mapping	Identification of unmet needs and gaps in care.
Process Mapping	Document existing workflows and pain points in mental health care.	Value Stream Mapping (Six Sigma)	Detailed understanding of inefficiencies.
Requirements Gathering	Define functional, ethical, and regulatory requirements for the platform.	Voice of the Customer (VOC)	Prioritized list of user-centric requirements.
Define Objectives	Establish clear project goals based on research findings.	SMART Goal Setting	Comprehensive and actionable project roadmap.
Journey Mapping	Create detailed user journeys to understand patient and clinician experiences across touchpoints.	Customer Journey Mapping	Visualized patient-clinician interaction pathways.
Baseline Metrics	Identify current metrics for quality, efficiency, and satisfaction as benchmarks for improvement.	KPIs, Statistical Analysis	Baseline data to measure project success.
Stakeholder Alignment	Facilitate workshops to align stakeholders on project vision and deliverables.	Cross-Functional Collaboration Tools	Consensus on shared goals and expectations.
Risk Analysis	Identify potential risks in conceptual design and mitigate them proactively.	Risk Matrix, SWOT Analysis	Reduced likelihood of delays and issues.

**Table 3:** Phase 2 - ideate and prototype (Framework Development).

**Step**	**Description**	**Methods**	**Key Outcomes**
Concept Ideation	Brainstorm potential solutions to address identified needs.	Design Thinking Ideation Sessions	Wide range of innovative ideas.
Rapid Prototyping	Low-fidelity prototypes of key platform features.	Wireframing, Mockups	Visual representation of potential solutions.
Process Design	Process improvements for clinical workflows.	DMAIC (Define, Measure, Analyze, Improve, Control - Six Sigma)	Optimized workflows ready for testing.
Feedback Iteration	User testing with prototypes and refinement based on input.	Scrum Sprints	Improved prototypes aligned with user needs.
Feasibility Assessment	Technical, operational, and financial viability of proposed solutions.	Feasibility Studies, SWOT Analysis	Selection of viable solutions for implementation.
High-Fidelity Prototyping	Advanced prototypes incorporating refined features and workflows.	Prototyping Software, Interactive Models	Realistic models for more effective stakeholder evaluation.
Integration Testing	Integration of prototypes with existing systems to assess compatibility and functionality.	Test Scenarios, API Testing Tools	Verified compatibility and functional readiness.

**Table 4 -:** Phase 3 - implement and validate (Pilot Testing).

**Step**	**Description**	**Methods**	**Key Outcomes**
Data Collection	Data was gathered on platform performance and user feedback.	Statistical Process Control (SPC)	Comprehensive performance metrics.
Risk Assessment	Identification and mitigation of risks during implementation.	Failure Mode and Effects Analysis (FMEA)	Reduced operational risks.
Validation	Ensure compliance with regulatory and quality standards.	Control Charts, Capability Analysis	Certified and validated platform readiness.
Training and Onboarding	Provide comprehensive training for clinicians and staff to maximize platform adoption.	Training Modules, User Manuals	Increased proficiency and effective usage.
Iterative Refinement	Feedback from users to refine platform features and workflows.	Scrum Sprints, Feedback Loops	Enhanced usability and alignment with user needs.
Performance Benchmarking	Established performance benchmarks to measure system efficiency and user satisfaction.	KPI Tracking, Benchmark Analysis	Clear performance indicators for system evaluation.
Compliance Audits	Regular audits to ensure ongoing compliance with ethical and regulatory requirements.	Audit Checklists, External Reviews	Continuous adherence to standards and guidelines.

**Table 5:** Phase 4 - scale and sustain (Expansion and Optimization).

**Step**	**Description**	**Methods**	**Key Outcomes**
Predictive Analytics	Integration of advanced features for early detection and intervention.	Machine Learning Models	Enhanced functionality for proactive care.
Stakeholder Engagement	Maintain collaboration with stakeholders for ongoing support and alignment.	Agile Stakeholder Reviews	Long-term stakeholder satisfaction.
Advanced Scalability	Developed modular frameworks to enable seamless scaling for larger infrastructures.	Scalable Architecture Design	Platform readiness for larger deployments.
Cross-Regional Testing	Future real-world testing in diverse geographical regions to validate adaptability.	Pilot Deployments, Regional Testing	Validated global applicability.
Ecosystem Integration	Integration with broader healthcare ecosystems, including wearable devices and third-party systems.	API Development, Interoperability Standards	Enhanced system compatibility and ecosystem reach.
Data Lifecycle Management	Advanced processes for data archiving, retrieval, and governance.	ISO/IEC Standards, Data Pipelines	Comprehensive and compliant data handling practices.

### 3.2 Phase 1: empathize and define (Conceptualization)

In the initial “Empathize and Define” phase, the project team focused on gathering comprehensive insights into current practices, unmet needs, and systemic inefficiencies in mental health and CNS disorder care. This included in-depth interviews, surveys, and focus group discussions with a broad range of stakeholders—clinicians, caregivers, policymakers, and IT specialists—whose combined perspectives illuminated recurring pain points. Design thinking’s empathy mapping techniques facilitated a clearer understanding of the users’ lived experiences, revealing workflow bottlenecks and fragmented interoperability that hindered data sharing and real-time clinical decision-making.

Concurrently, value stream mapping (derived from Six Sigma) pinpointed inefficiencies such as excessive administrative tasks and delayed interprofessional communication, highlighting opportunities for automation and AI-driven solutions. This analytical stage allowed the project team to define technical, clinical, and user-experience requirements, ensuring alignment with GDPR, the EU AI Act, and other regulatory frameworks. Objectives were set according to SMART (Specific, Measurable, Achievable, Relevant, Time-Bound) criteria, establishing quantitative baseline metrics, including diagnostic turnaround times, clinician workload, and patient adherence rates. To anchor these insights in a coherent project roadmap, stakeholder alignment workshops were organized to validate the proposed direction and manage key risks—such as anticipated resistance to novel technologies and potential data security vulnerabilities—through structured analyses (e.g., SWOT, risk matrices). By balancing empathy-driven research and rigorous quality assurance, this first phase laid the conceptual foundation for a platform that could effectively integrate clinical decision support with mental health interventions.

### 3.3 Phase 2: ideate and prototype (framework development)

Building on the conceptual groundwork, Phase 2— “Ideate and Prototype”—focused on shaping the SMILE platform’s features and workflow designs into tangible, testable models. Drawing on Agile and Scrum methodologies, the project team worked in short, iterative sprints, refining ideas through continuous feedback loops. Collaborative brainstorming sessions brought together clinicians, engineers, data scientists, and user-experience experts to explore innovative solutions for the challenges identified in Phase 1, such as complex care pathways and limited personalization of therapeutic resources. Low-fidelity prototypes (wireframes, mockups) enabled stakeholders to visualize proposed solutions early in the design process. Feedback derived from quick usability tests and structured design thinking workshops accelerated iterative improvements, ensuring that evolving prototypes remain clinically relevant and user-friendly. Concurrently, Six Sigma’s DMAIC (Define, Measure, Analyze, Improve, Control) framework was employed to optimize underlying processes—ranging from automated clinician-patient communication to AI-driven decision support—while maintaining alignment with efficiency and regulatory standards. Integration testing at this stage confirmed the feasibility of connecting prototype modules with existing healthcare systems, such as electronic health records, highlighting both the platform’s technical viability and compliance with critical data protection guidelines.

### 3.4 8D analysis methodology

**Fig. 4:**
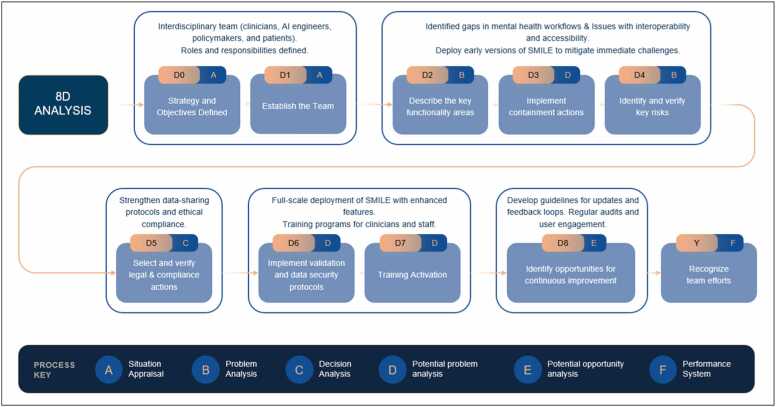
Structured 8D analysis problem-solving for SMILE implementation.

Throughout prototyping, the team adopted an 8D problem-solving approach (Fig. 4) to diagnose recurring issues, implement corrective measures, and establish preventive strategies. Initially, the interdisciplinary group defined problems tied to interoperability constraints and user adoption barriers, promptly implementing interim fixes to stabilize pilot features. Root cause analysis followed, revealing deeper infrastructural and process-level inefficiencies. Permanent corrective measures—such as refining data exchange protocols and incorporating user-friendly design principles—were then executed. Finally, preventive actions were developed to ensure the sustainability of solutions and to support future enhancements. Recognizing the contributions of diverse stakeholders after each 8D cycle reinforced a collaborative culture crucial for Phase 3 and beyond.

### 3.5 Phase 3: implement and validate (pilot testing)

The transition from prototyping to real-world application occurred in Phase 3, where the SMILE platform was deployed across varied clinical contexts, including outpatient clinics, hospital neurology departments, and community mental health centers. This careful site selection aimed to capture a breadth of user experiences and operational workflows, thus providing a robust basis for evaluating both platform scalability and clinician acceptance.

Pilot implementations were governed by Agile frameworks, with incremental releases and scheduled Scrum iterations. Statistical process control methods enabled the systematic monitoring of key performance metrics, such as patient engagement levels and clinician satisfaction. Simultaneously, Failure Mode and Effects Analysis (FMEA) was employed to identify and mitigate risks, offering a structured approach to preserving data security, functionality, and compliance with regulations like GDPR and ISO/IEC 27001. Training sessions played a pivotal role in promoting platform adoption, focusing on both technical proficiency and IDC, thereby empowering users to integrate SMILE’s AI-driven insights into routine clinical decision-making. Quantitative data—encompassing turnaround times, diagnostic accuracy, and system uptime—were complemented by qualitative evidence from user interviews, on-site observations, and survey instruments. Comprehensive validation procedures confirmed that the platform’s adaptive CBT modules, NLP-based interventions, and consent management features performed reliably under real-world conditions, while regular compliance audits verified adherence to ethical and regulatory standards.

### 3.6 Root cause analysis (RCA) map

**Fig. 5:**
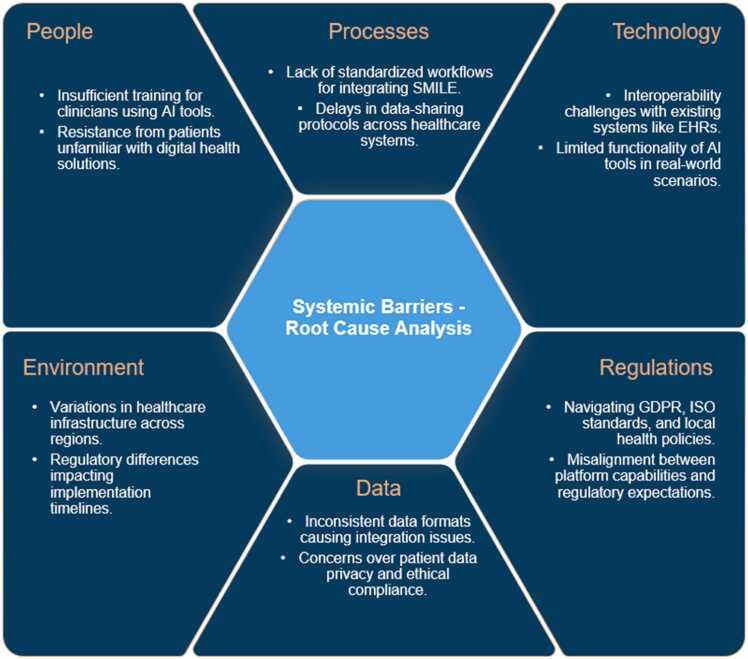
Identifying systemic barriers - root cause analysis.

To systematically diagnose implementation barriers, a fishbone (Ishikawa) diagram (Fig. 5) organized challenges into six domains: people, processes, technology, environment, data, and regulations. This classification revealed that “people” issues, such as inadequate training and skepticism toward AI tools, intersected with “process” inconsistencies in workflow design. “Technology” categories underscored interoperability hurdles and AI software constraints, while the “environment” dimension encompassed infrastructural challenges and resource limitations. Under “data,” formatting inconsistencies and privacy concerns emerged, and “regulations” highlighted the complexity of cross-jurisdictional compliance. By illuminating these interconnected factors, the RCA map facilitated targeted interventions and contributed to an improved alignment between the SMILE system’s design and healthcare practitioners’ practical realities.

### 3.7 Phase 4: scale and sustain (expansion and optimization)

With successful pilot testing providing validated outcomes, the SMILE project advanced to the “Scale and Sustain” phase, focusing on broadening its impact and ensuring enduring efficacy. Lessons from pilot sites were codified and translated into platform refinements, including the extension of multilingual capabilities and the incorporation of region-specific regulations to facilitate global reach. Enhanced predictive analytics modules—targeting the early detection of sporadic mental disorders—were introduced, broadening the platform’s clinical applicability. Cross-regional testing further validated SMILE’s adaptability in settings that ranged from low-resource clinics to large academic medical centers. Coordination with local IT infrastructures, as well as adherence to recognized standards such as HL7 FHIR, underpinned successful ecosystem integration. By offering application programming interfaces and adhering to interoperability frameworks, the platform ensured a seamless flow of data across various healthcare services, including wearable devices and complementary mental health tools. Continuous improvement principles anchored this phase, with iterative updates driven by stakeholder feedback and new technological developments. Meanwhile, data lifecycle management—encompassing archiving protocols, retrieval systems, and governance policies—was rigorously aligned with GDPR and ISO/IEC 27001 to safeguard patient privacy. Furthermore, partnerships with government organizations, nonprofits, and industry players secured funding and bolstered the SMILE platform’s presence on the global healthcare stage, ensuring long-term viability.

### 3.8 Phase 5: evolve and innovate - continuous evolution

Finally, the “Evolve and Innovate” phase established an enduring framework for ongoing enhancement of the platform. Collaboration and co-creation remained central tenets of this phase, exemplified by expanded feedback loops that included patient advocacy groups, academic institutions, and technology start-ups. This ecosystem-based model has stimulated fresh innovations in mental health interventions and fostered an inclusive community. The emphasis on ethical considerations has extended to sustainability, equity, and governance. By aligning operational practices with environmental, social, and governance principles, the platform underscores a long-term commitment to ethical healthcare innovation. The modular, scalable architecture has proven capable of responding swiftly to emergent challenges, including public health crises and environmental disasters with psychosocial repercussions. This adaptability ensured that the platform remains both clinically relevant and operationally robust, prepared to meet the future demands of mental health care. By integrating continuous innovation, global collaboration, and conscientious social responsibility, the SMILE initiative concludes its developmental roadmap positioned as a transformative solution for both immediate and long-term needs in digital health.

### 3.9 Architecture: integrating learning, identity management, and AI-driven support

The platform supports the augmentation of an existing point-of-care decision support system while also serving as an educational resource within a learning management system or continuing medical education platform. Unlike traditional stand-alone CDSS, SMILE weaves together three fundamental elements—behavioral training resources, secure identity and data management, and AI-driven mental health support—into a coherent architecture. Its design focuses equally on safeguarding personal information, conforming to European data protection regulations, and delivering training and stress management interventions for HCPs.

Through standardized data formats and interoperable application programming interfaces, SMILE’s modules can be embedded directly into the interfaces clinicians use every day. This reduces adoption barriers, allows for real-time updates on best practices, and ensures consistency in care. Furthermore, the learning environment aspect lets SMILE deliver regular content updates informed by the latest clinical guidelines, making it easier for HCPs to remain current on evidence-based treatments for both mental health concerns and broader CNS disorders. A peer support feature, bolstered by machine learning techniques, allows HCPs to connect in a confidential environment, share experiences, and offer mutual assistance. SMILE’s algorithms can triage requests by urgency, funneling critical cases to appropriate mental health resources or trained colleagues for timely intervention. This fosters a community of practice that alleviates feelings of isolation, a key contributor to burnout in high-pressure healthcare settings. To address the heightened sensitivity of mental health-related information, SMILE employs blockchain for identity management. Users, whether clinicians or nurses are assigned unique decentralized identifiers managed under a self-sovereign identity framework. This ensures that individuals have granular control over their data, selectively disclosing only the portions pertinent to training, peer support, or clinical decision-making.

On-chain smart contracts automate access policies, audit trails, and compliance enforcement. Whenever a clinician seeks to review a peer’s shared resource or personal performance data, a hashed record of the access request is appended to the permissioned blockchain ledger. This tamper-evident method meets stringent regulatory requirements and reassures stakeholders that mental health information remains confidential and properly monitored. Additionally, end-to-end encryption and a zero-trust architecture protect data at rest and in transit, ensuring robust safeguards against unauthorized intrusions and insider threats. To preserve user data privacy and compliance with GDPR, federated learning protocols allow the SMILE model to continuously refine its capabilities without aggregating raw clinician data in a central repository. Participating nodes (e.g., local hospital servers) share model gradients rather than actual data, thereby reducing the likelihood of data breaches while simultaneously advancing the system’s performance across diverse clinical environments. SMILE’s support and educational modules often require large datasets to train AI components—particularly those related to predictive algorithms for stress management. In compliance with data minimization principles, the platform employs generative models like generative adversarial networks and variational autoencoders for synthetic data creation. These artificially generated datasets mirror the statistical properties of authentic mental health data, allowing iterative model improvements without risking patient or clinician re-identification. By iterating between generator and discriminator networks, generative adversarial networks produce high-fidelity synthetic samples closely resembling real-world mental health scenarios. This is particularly useful for simulating complex or rare clinical presentations. Regarding variational autoencoders, they capture the variability and subtleties of mental health data by encoding and decoding samples in a latent space. They generate diverse training examples that reflect a wide array of therapeutic contexts, helping the platform adapt to heterogeneous user needs. Noise injection or anonymization procedures further protect sensitive attributes in both real and synthetic datasets. These steps ensure compliance with GDPR’s principle of data protection by design, fostering trust among clinicians and institutions.

Additionally, throughout its deployment, SMILE integrates ethical safeguards and transparent governance measures. Informed consent protocols delineate data usage, while routine audits verify adherence to privacy rules and confirm the platform’s integrity. Explainability is emphasized, as clinicians and administrators can review documentation about SMILE’s synthetic data pipelines, AI-based recommendations, and identity management workflows. Such transparency fosters confidence among stakeholders and aligns with evolving legislation and guidelines from bodies like the European Data Protection Board. By uniting a blockchain-based identity framework, AI-driven mental health support, and comprehensive educational tools, SMILE stands poised to transform how HCPs learn, practice, and maintain their well-being. Its architecture is designed around an ethos of trust, privacy, and adaptability, ensuring that clinicians can confidently adopt new technologies, remain updated on best practices, and receive individualized support—free from stigma and organizational barriers. This multifaceted approach not only bolsters clinical effectiveness but also nurtures a more humane and sustainable healthcare environment, ultimately enhancing patient care and fostering enduring improvements in mental health outcomes for both practitioners and the populations they serve.

**Fig. 6:**
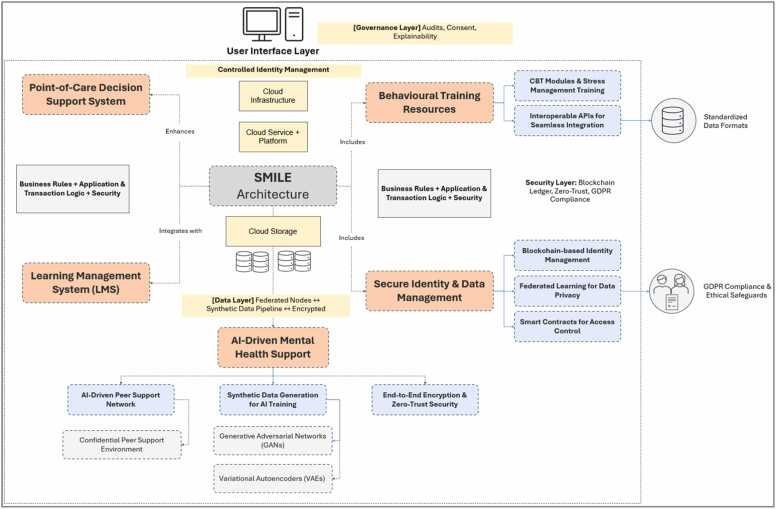
Identifying systemic barriers - root cause analysis.

Fig. 6 depicts the SMILE platform’s multi-layered design using four distinct colors to convey the relationships and functions of its components. Orange blocks represent the core functional elements, including the Point-of-Care Decision Support System, the Learning Management System, Behavioral Training Resources, and the Secure Identity & Data Management module. These elements form the principal pillars of SMILE: they supply clinical decision-making capabilities, user education, and mechanisms for handling privacy and identity verification. At the center, in grey, lies the SMILE Architecture, which integrates and coordinates the core orange elements. This grey block governs transaction logic, security protocols, and application rules—ensuring that clinicians receive unified workflows and secure, compliant data handling regardless of which component they access. Surrounding this central architecture is the yellow “Data Layer,” indicating that data storage, federated nodes, and synthetic data pipelines function as foundational supports for SMILE’s overall operation. Federated learning occurs here, allowing each node to train AI models locally while avoiding centralizing sensitive clinical information. Encryption and additional data protection measures align with GDPR requirements, preserving privacy and adherence to ethical principles. Finally, light blue sections illustrate AI-Driven Mental Health Support modules. These include the AI-driven peer support network and synthetic data generation tools, such as Generative Adversarial Networks and Variational Autoencoders. Placing these features in light blue differentiates them as specialized, advanced AI functionalities. They provide real-time monitoring, stress management resources, and robust mental health interventions for HCPs, reinforcing SMILE’s focus on well-being. Overall, the core systems, orchestrating architecture, foundational data layer, and specialized AI tools interlock to deliver a holistic solution that enhances clinical decision support, fosters ongoing professional development, and upholds stringent data privacy standards.

## 4 Results

### 4.1 Demographic characteristics, relationships and preliminary findings

In the pilot phase, the platform was introduced to clinicians operating within distinct clinical environments across Germany (6 participants), Switzerland (5 participants), the United States (5 participants), Canada (5 participants), and Portugal (5 participants). This multinational selection was intended to capture a broad spectrum of workflow variations and resource constraints. Participants were initially familiarized with the platform’s user interface and privacy features, after which they submitted baseline stress measurements, time spent seeking clinical support, and overall satisfaction. Over a period of six months, changes were monitored in these metrics as HCPs increasingly incorporated their automated alerts, interactive CBT prompts, and blockchain-enabled consent management into routine practice. Regular feedback sessions allowed the development team and local coordinators to refine modules, enhance user engagement, and address technical obstacles. Emphasizing anonymity and confidentiality was imperative, given that some HCPs were wary of potential risks associated with mental health disclosures in a professional setting. Importantly, the data employed for testing remained anonymized and generated specifically for controlled evaluation scenarios, relying on Likert-scale responses for pre- and post-implementation comparisons.

Although SMILE has thus far been restricted to pilot deployment in outpatient clinics, hospital neurology units, and community mental health centers, its ongoing transition from prototype to larger-scale implementation is still under review. This strategic site selection aimed to identify a wide array of user experiences, thereby establishing a robust basis for assessing the platform’s scalability and clinician acceptance. The pilot enrolled 26 HCPs, resident physicians, allied health practitioners, and administrative staff—from diverse demographics. The average age was thirty-nine, and approximately 65 % were female. Nearly half of the participants were in direct patient-facing roles, managing tasks such as triage, medication management, and psychosocial support. Another portion, primarily physicians and specialized clinicians assumed responsibilities for more complex diagnostic evaluations.

All participants underwent targeted training on SMILE’s functionalities, including CBT-based personal well-being tools, step-by-step prompts for intricate clinical scenarios, and real-time guidance for best practices. Baseline data—encompassing stress (1–10 scale), satisfaction (1–10 scale), and perceived value—were acquired before the platform’s integration, followed by reassessments four weeks later. Preliminary analyses indicated that the observed improvements were both statistically and practically significant. Three salient trends emerged from these data. First, HCPs reported a reduction in “support time,” suggesting that the platform’s consolidated resources and single sign-on method expedited information retrieval. Second, a decrease in stress levels was observed, with participants attributing this shift to streamlined workflows and SMILE’s mental health support tools (e.g., self-assessments and CBT modules). Third, substantial increases in satisfaction and perceived value implied that users not only benefited from efficiency gains but also recognized the platform’s importance to their professional duties.

Qualitative feedback further revealed that any perceived improvements in efficiency were linked to participants’ trust in SMILE’s reliability and ethical handling of sensitive information. Although several HCPs initially voiced apprehensions regarding data confidentiality, these concerns were largely alleviated once they engaged with the platform’s encryption protocols and blockchain-based identity management features. Surveys indicated that many users were motivated to continue using SMILE by the reassurance that personal stress indicators and self-assessment results would remain confidential. Overall, the pilot underscored a pivotal development in healthcare: clinicians require integrated systems that address both process-driven optimizations and their mental well-being. Despite the modest sample size, the findings underscore the necessity for continued evaluations on a broader scale. Reduced support times and lower stress levels have the potential to translate into more consistent patient care, while enhanced satisfaction and perceived value may alleviate turnover among stressed clinical staff. Such evidence supports SMILE’s potential to bridge multiple gaps: it addresses the substantial need for improved mental health support among HCPs while simultaneously mitigating inefficiencies in clinical workflows.

**Fig. 7:**
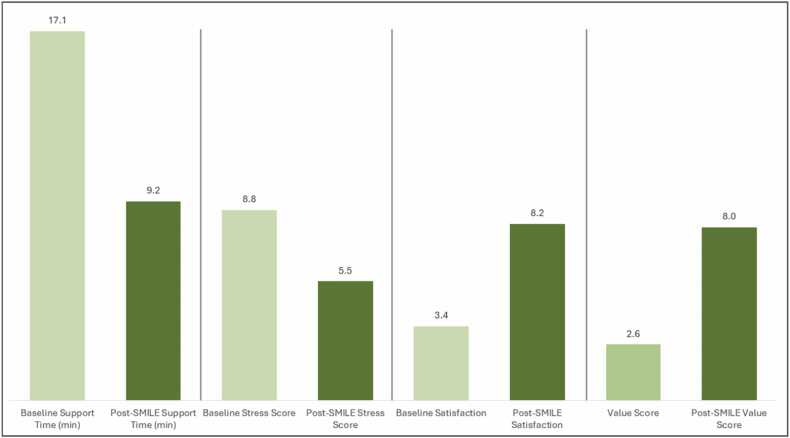
Support, stress, satisfaction, and value score.

At the outset, the pilot’s twenty-six participants captured a range of clinical roles, schedules, and stress exposures, producing a representative initial sample. By integrating both quantitative (e.g., baseline vs. post-SMILE metrics) and qualitative data (e.g., focus group insights), the study enabled an in-depth analysis of how the platform influenced everyday practice. Indeed, the bar chart in Fig. 7 highlights the intervention’s effects on four principal variables: support time, stress, satisfaction, and value. From baseline to post-SMILE, support time declined from 17.1 to 9.2 min, and stress levels fell from 8.8 to 5.5, while satisfaction and value scores increased from 3.4 to 8.2 and from 2.6 to 8.0, respectively. Participants initially struggling with high stress and increased reliance on support tended to show the largest improvements. The combination of CBT-focused modules, an intuitive user interface, and peer-based support channels appears to have mitigated core pain points. The overall reduction in stress, alongside lower support times, points to SMILE’s potential for addressing the cyclical relationship between limited resources and elevated clinician burnout. By creating more direct channels for knowledge-sharing, decision support, and self-care, SMILE demonstrates a tangible capacity for improving both procedural efficiency and user well-being within diverse healthcare settings.

### 4.2 Statistical analysis

Cronbach’s alpha values of approximately 0.40 were observed for the stress, satisfaction, and value items, indicating moderate internal consistency. Although a threshold of 0.70 is often cited to designate acceptable unidimensional scales, these results suggest that stress, satisfaction, and value might represent distinct facets of participant experiences rather than a single cohesive construct. Examining standard deviations further highlighted shifts in variability. For instance, the baseline support time (SD = 3.99) exceeded the post-SMILE support time (SD = 2.42), suggesting a narrowing dispersion once the intervention was in place. Similarly, post-SMILE stress and satisfaction exhibited more uniform distributions, implying that most participants converged toward lower stress and higher satisfaction. By contrast, the post-SMILE value score maintained a relatively broad spread (SD = 1.39), possibly reflecting individual or role-related factors shaping user perceptions.

A detailed comparison of pre-and post-intervention metrics underscored sizable changes in satisfaction (an increase of approximately 4.7 points) and perceived value (around 5.4 points). Although stress and support time displayed somewhat smaller yet still statistically robust changes, these findings confirm that SMILE’s implementation coincided with meaningful improvements in core clinical and personal metrics. To evaluate the statistical robustness of these outcomes, paired sample t-tests were performed on each variable, revealing significant reductions (p < 0.0001) for both support time and stress, along with notable increases in satisfaction and value. Effect sizes (Cohen’s d) were substantial, underscoring the practical relevance of these shifts. An ANOVA approach corroborated the reliability of these differences across the participant sample, with high F values (e.g., F=74.30 for support time) and p-values near zero, further negating the likelihood that random variation explains the observed changes.

Correlation analyses highlighted inverse relationships, for example, between post-SMILE stress and satisfaction—as well as direct associations, such as between post-SMILE value and satisfaction. A linear regression model examining post-intervention variables accounted for a modest 1.4 % of the variance in satisfaction (R² = 0.014), indicating that other, unmeasured factors may also shape user satisfaction. Nonetheless, the strong significance levels, accompanied by robust effect sizes, underscore the capacity of SMILE to substantially reduce support time and stress while enhancing satisfaction and perceived value. Taken together, these quantitative findings offer a strong empirical basis for the pilot’s claim that SMILE effectively supports HCPs by alleviating workload pressures and facilitating more positive user experiences, meriting larger-scale follow-up studies.

Table 6 shows that after the SMILE intervention, participants spent less time requiring support (dropping from an average of 17.08 min to 9.19 min) and experienced lower stress (from 8.81 down to 5.50). At the same time, both satisfaction and perceived value rose substantially: satisfaction scores increased from 3.42 to 8.15, and value scores went from 2.58 to 8.00. All of these changes have narrow 95 % confidence intervals for the mean differences—ranging between about 6 and 9.5 min for support time, about 2.8 and 3.7 for stress, 4.4 and 5.0 for satisfaction, and 5.0 and 5.6 for value—indicating a statistically significant shift in each metric from baseline to post-SMILE.

**Table 6:** Descriptive statistics for key metrics (Baseline vs. Post-SMILE).

**Metric**	**Baseline (Mean ± SD)**	**Post-SMILE (Mean ± SD)**	**95 % Confidence intervals for Difference**
Support Time (min)	17.08 ± 3.99	9.19 ± 2.42	[5.96, 9.53]
Stress Score (1–10)	8.81 ± 1.10	5.50 ± 1.07	[2.83, 3.70]
Satisfaction (1–10)	3.42 ± 1.27	8.15 ± 1.22	[4.38, 5.03]
Value Score (1–10)	2.58 ± 1.27	8.00 ± 1.39	[5.01, 5.63]

### 4.3 Correlation heatmap analysis

**Fig. 8:**
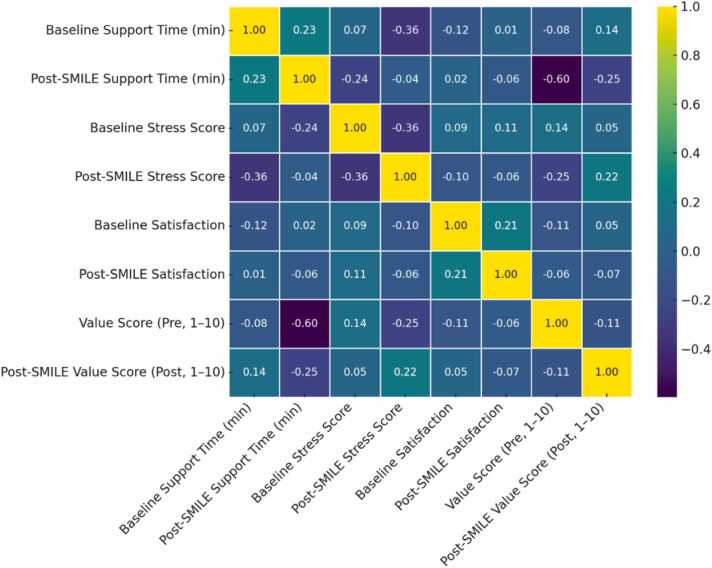
Heatmap of key dimensions and correlations.

Fig. 8 offers important insights into the interrelationships among key variables, both before and after the intervention. By mapping numerical correlation values onto a gradient spanning teal (indicative of strong negative correlation) to yellow (strong positive correlation), this visualization streamlines the interpretation of complex statistical associations. Although each correlation should be interpreted within the broader context of diverse clinical environments, the heatmap nonetheless provides a succinct overview of how support time, stress levels, satisfaction, and perceived value intersect. One notable observation is the positive correlation of approximately 0.23 between Baseline Support Time (min) and Post-SMILE Support Time (min). Although 0.23 does not signify a particularly strong correlation, especially compared to more substantial coefficients typically seen in other studies—it implies that clinicians requiring relatively high support time at baseline remained those who needed more support post-intervention, even though all participants generally benefited from reduced support durations (e.g., from a mean of 17–9 min). These findings underscore SMILE’s capacity to lower total support times without eliminating individual variations in resource needs. Future updates might focus on tailoring interventions for high-need users to further decrease their support times.

Another salient result is the modest negative correlation (around −0.10) between Baseline Stress Score and post-SMILE satisfaction. Although the coefficient is small and does not necessarily establish causation, it suggests that HCPs with elevated stress at baseline tended to exhibit greater satisfaction post-intervention. These individuals may have perceived SMILE’s CBT modules and streamlined workflows as especially beneficial, leading to more pronounced gains. In contrast, Post-SMILE Stress Score demonstrates weaker correlations overall (e.g., −0.36 relative to Baseline Support Time and −0.25 relative to pre-intervention Value Score), indicating that multiple factors—ranging from the extent of CBT module usage to personal resilience strategies—likely moderate stress reduction outcomes.

The heatmap also reveals a positive correlation (roughly 0.21) between Baseline Satisfaction and post-SMILE satisfaction, suggesting that initial attitudes toward digital interventions can persist, even as satisfaction evolves significantly (e.g., from an average of 3.42–8.15). This result implies that while SMILE yields improvements, baseline perceptions can either amplify or dampen its immediate impacts. Similarly, the correlation between Value Score (Pre, 1–10) and Post-SMILE Support Time (min) hovers around −0.60, reflecting one of the more pronounced negative correlations and indicating that individuals who initially placed higher value on available tools tended to show more marked reductions in post-intervention support time.

Factors such as institutional culture, personal coping mechanisms, or external stressors may also influence the observed relationships. As an example, the relatively low correlation of 0.09 between Baseline Stress Score and Baseline Satisfaction may reflect the reality that some clinicians manage stress effectively despite high-pressure roles, while others’ satisfaction is deeply affected by stress. Likewise, the near-zero correlation (0.02) between Baseline Satisfaction and Post-SMILE Support Time implies that changes in support time are not necessarily tied to initial satisfaction levels. Participants who initially needed extensive support tended to remain in that higher-support cohort post-intervention, albeit with shorter durations overall. Those experiencing heightened stress at baseline often reported substantial gains in post-SMILE satisfaction, and users with positive pre-existing attitudes toward available resources tended to incorporate SMILE’s features more efficiently. Consequently, tailoring user onboarding and support strategies based on these varied predispositions may optimize outcomes and further strengthen SMILE’s capacity to enhance clinician well-being and professional efficiency.

## 5 Discussion

### 5.1 Key collected themes, identified patterns, and insights

Drawing on both quantitative metrics (e.g., support time, stress levels, satisfaction, and value scores) and qualitative feedback (e.g., focus groups, user comments), prominent themes emerged that illuminate the platform’s potential contributions and its areas of vulnerability. One of the most salient insights was the critical role of user-centric design in supporting adoption and continued engagement. From the earliest phases of conceptualization through prototype refinement, stakeholders consistently highlighted the importance of intuitive navigation, customizable dashboards, and context-sensitive prompts—particularly when dealing with time-sensitive care settings and high-stress environments. In practice, participants who reported a more seamless integration of SMILE’s tools into their daily workflows were also those who showed greater reductions in both support time and self-reported stress. This intersection between platform usability and measurable outcome improvements underscores the potential for carefully tailored digital interventions to facilitate clinicians’ tasks.

A second key pattern revealed itself in the relationship between personal stress levels and the perceived benefit of the SMILE modules, such as CBT and NLP-based supportive tools. Participants entering the pilot with higher self-reported stress scores often experienced the most pronounced decrease in stress by the conclusion of the study period. The qualitative data from interviews and post-simulation sessions suggested that clinicians who were initially close to burnout thresholds found significant value in SMILE’s capacity to manage routine administrative burdens and offer emotional support through peer forums. These findings lend credence to the platform’s premise of augmenting clinical decision support with structured mental health management interventions, highlighting that improvements in workflow efficiency can go hand in hand with enhanced emotional resilience.

Furthermore, the theme of adaptive learning and professional development emerged as integral to SMILE’s user appeal. The pilot revealed that clinicians are more likely to trust and rely on digital platforms when these platforms incorporate up-to-date clinical guidelines, ongoing professional development modules, and easy-to-access references that promote evidence-based practice. Because SMILE integrates real-world knowledge updates (e.g., new CBT techniques and updated treatment guidelines for depression and anxiety), participants perceived it not only as a clinical tool but also as a continuous learning resource. This synergy is particularly pertinent in fast-evolving fields like mental health, where new therapies and research findings frequently alter best practices.

The importance of data privacy and secure identity management also featured prominently. By leveraging blockchain for consent tracking and decentralized identity solutions, SMILE sought to build trust with both HCPs and patients. Qualitative comments underscored that data-sensitive participants—often those most anxious about potential security breaches—were more at ease engaging with SMILE once they understood the platform’s encryption protocols, zero-trust architecture, and granular access controls. The successful reduction of data-sharing concerns, as evidenced by lower reported anxiety about digital record-keeping, further validated the approach of employing advanced security frameworks within an AI-driven tool. Meanwhile, efficiency gains in clinical workflows resonated across multiple data points. The observed reduction in “support time” was widely associated with SMILE’s capacity to aggregate guidelines, incorporate patient data into clinically relevant dashboards, and prompt timely interventions. Participants specifically noted that standardized care pathways and checklists eased the burden of repetitive tasks, while integrated CBT prompts and self-assessment tools helped them remain mindful of their mental health. These efficiency gains imply that adopting such integrated solutions can address not just patient-facing workflows but also the well-being of providers, supporting the notion that a more organized and responsive system fosters greater job satisfaction.

A final insight concerns heterogeneous adoption patterns. While the pilot data illuminated an overall positive shift in user attitudes and outcomes, variations among individual participants highlight the need for nuance. Clinicians with prior exposure to telehealth or AI-based platforms typically navigated SMILE’s features and reaped the benefits more swiftly than those less technologically savvy. Additionally, differences in organizational culture, leadership support, and resource allocation influenced how quickly staff adapted to the new workflow processes. This variation suggests that broader, system-wide integration of SMILE would benefit from structured onboarding, role-based training modules, and a clear articulation of organizational expectations and support mechanisms. The key patterns and insights gleaned from the pilot reinforce the platform’s potential for meaningful impact on mental health care while also underscoring the complexity of implementing a digital solution in diverse clinical and psychosocial landscapes. By zeroing in on user-centric design, data security, educational resources, and supportive workflows, SMILE has outlined a strategy for bridging the gap between technological innovation and practical healthcare needs.

### 5.2 Comparison of findings with previous literature

Situating SMILE’s outcomes within the broader body of mental health and digital health research reveals notable points of convergence and differentiation. Over the past decade, multiple studies have underscored the rising importance of AI-driven decision support systems and the concomitant ethical and practical implications of deploying them in clinical environments. The pilot results for SMILE align with earlier findings that highlight how AI-augmented platforms can reduce clinician workload and potentially mitigate burnout. Yet SMILE’s multifaceted approach—combining an educational LMS component, active peer support channels, and advanced identity management via blockchain—adds a fresh dimension to this line of inquiry [28].

Studies on electronic health record optimization and physician burnout have shown that poorly designed digital solutions can exacerbate clinician fatigue, primarily due to cognitive overload and administrative complexities. In contrast, the SMILE pilot participants reported a consistent drop in the required time and a substantial jump in satisfaction scores. These results parallel the consensus in literature that thoughtfully integrated solutions (i.e., single sign-on, streamlined dashboards, automated alerts) are more likely to succeed. A growing corpus of psychological and occupational health studies underscores the significance of structured interventions, particularly CBT-based approaches—for HCPs navigating chronic work stress. SMILE’s focus on embedding CBT modules directly into the clinical workflow for HCPs is a step beyond typical telehealth or mental health apps that primarily target patient populations. This aligns with recent publications suggesting that addressing clinicians’ mental well-being is integral to optimizing patient care quality. Consequently, SMILE’s synergy of organizational, clinical, and well-being features positions the system at the forefront of integrated mental health solutions, whereas previous literature often treats these domains separately [22], [30].

The growing momentum of blockchain in healthcare is frequently cited for its potential to advance security, transparency, and patient agency. SMILE’s pilot echoes these advantages, where participants reported greater confidence that their data or self-assessments would remain private. However, the actual adoption of permissioned ledgers within large-scale health systems remains nascent, as prior studies highlight logistical barriers such as transaction throughput and stakeholder willingness to adapt legacy systems. Similarly, the use of federated learning for preserving data privacy aligns with emergent themes in big data literature, where distributed training models have been validated in collaborative research networks (Sheller et al., 2020). Also, SMILE’s pilot, by showcasing a feasible integration of federated learning with clinical workflows, reinforces these earlier results, suggesting that privacy-preserving analytics can be deployed even in sensitive clinical contexts like mental health [19], [27].

Comparisons with other AI-driven interventions confirm the tension between rapid technological advances and the slow pace of regulatory standardization. Studies focusing on the EU AI Act and GDPR underscore the intricacies of data governance, an area that SMILE also grapples with, given its cross-border ambitions and its usage of personal mental health data. The pilot’s emphasis on transparent data-handling protocols, real-time consent tracking, and robust encryption aligns with best practices championed by health informatics researchers in Europe and beyond. However, the literature points out a consistent gap in applying such frameworks to resource-limited settings or developing countries with less infrastructure. SMILE’s approach of partial offline or low-bandwidth adaptation is, therefore, an innovative extension, but it remains to be seen if these adjustments fully align with the complexity of regulatory compliance in varied regions [16].

Where SMILE diverges from some of the literature is in the explicit pairing of a functional CDSS with an ongoing professional development system, culminating in a more holistic approach to mental healthcare for both patients and clinicians. While the current pilot data is consistent with the notion that integrated solutions yield better user adoption and positive attitudes, further investigation across multiple institutions and countries would provide stronger validation. Moreover, the pilot’s reliance on modest sample size is reminiscent of pilot studies in telehealth and e-mental health domains, which caution that initial results may be inflated by participant enthusiasm and targeted recruitment [35]. SMILE’s findings correlate strongly with existing empirical literature emphasizing user-centered design, privacy-preserving AI, and CBT-based resilience frameworks. Its unique contributions revolve around the synergy of these elements in a unified platform that addresses the mental health needs of HCPs, an area previously treated as ancillary to patient-facing digital interventions. The pilot data thus enrich existing debates on digital transformation in healthcare and open avenues for more rigorous, large-scale comparative studies [11].

### 5.3 Ethical and legal landscape

In navigating the complex domain of mental healthcare technology, SMILE presents a paradigm that underscores both the opportunities and the challenges inherent in adopting AI-driven, data-centric interventions. The pilot findings confirm the potential for improved user satisfaction, reduced stress, and heightened efficiency, but these outcomes do not diminish the extensive ethical and legal considerations that accompany advanced digital platforms. The sensitivity of mental health data intensifies the demand for meticulous privacy protections, a reality that SMILE addresses via blockchain-based identity management and permissioned ledgers. In principle, the “self-sovereign identity” approach grants users (e.g., HCPs or patients) granular control over how and when their data is accessed, better aligning with privacy mandates under GDPR. By logging each request onto the blockchain, SMILE bolsters traceability and fosters trust among stakeholders who may be concerned about potential misuse of personal health data. Yet, as the pilot was confined to a limited and technologically equipped environment, the question remains whether these frameworks can consistently function in broader, more heterogeneous networks where infrastructural gaps or conflicting regulations exist [35].

Even though the pilot indicated positive shifts in stress and support times, it also surfaced the necessity of algorithmic audits and fairness metrics. Large language models can inadvertently propagate biases, especially if trained on datasets with demographic skews. The portion of the pilot population with more specialized roles (e.g., advanced practice clinicians) or from minority groups reported uncertainty about whether the system’s recommendations fully accounted for their unique professional contexts. This points to the risk of underserving certain user subgroups—a concern that resonates with broader critiques of AI systems in healthcare, where disparities in data representation can lead to uneven care quality [25].

SMILE aspires to scale internationally, thereby confronting a tapestry of disparate legal frameworks. While the European Union’s regulatory landscape is shaped by GDPR and incipient regulations such as the EU AI Act, other regions maintain different, and sometimes conflicting, data protection laws or healthcare statutes. The pilot’s partial compliance with recognized standards like HL7 FHIR R4 and ISO/IEC 27001 is laudable, but cross-border expansion will require active collaboration with local authorities to ensure that blockchain transactions, identity solutions, and AI modules align with region-specific guidelines. Additionally, the interplay of multiple regulatory bodies raises the specter of overlapping or contradictory mandates, complicating matters like data portability and user consent.

From an ethical standpoint, the global scale-up of SMILE highlights questions of equitable access and the risk of exacerbating existing healthcare disparities. Regions with limited bandwidth, inconsistent electricity, or outdated hardware may struggle to implement advanced AI processes or store secure ledger entries on a blockchain. Such infrastructure challenges could leave underserved populations outside the scope of SMILE’s benefits. Ethically, it would be incumbent on the platform’s developers and sponsoring organizations to propose tiered or offline-ready models (e.g., compressed AI modules, satellite-based internet solutions) that mitigate these barriers. The pilot demonstrates feasibility under favorable conditions, but real-world deployments in lower-resource areas would require significant technical, financial, and policy adjustments to maintain compliance and user trust. In discussing liability and accountability, mental health interventions carry added weight. Although SMILE focuses on clinicians’ mental well-being, patients’ data can also become part of the system through integrated healthcare records or collaborative care modules. Here, the principle of informed consent must remain paramount. The pilot’s consenting processes were suitably robust for a small-scale test, but scaled rollouts require ensuring every patient or clinician fully comprehends how their data journeys through advanced AI modules and decentralized identity networks. Achieving genuine autonomy may entail translating policies into multiple languages, employing plain-language explanations of data usage, and providing easy pathways to opt-out.

Against these complexities, SMILE’s layered approach—emphasizing peer support, blockchain-based data control, and continuous engagement with regulatory shifts—offers a blueprint for responsibly advancing AI in mental health contexts. Nevertheless, the pilot underscores the non-trivial effort required to maintain compliance, address algorithmic fairness, and respect the autonomy of both clinicians and patients. As the platform moves toward broader or more diverse environments, embedding an adaptive legal and ethical monitoring framework is essential to sustain trust and scale effectively.

### 5.4 Future work

While the pilot data unequivocally point to the SMILE platform’s promise of optimizing mental health care and reducing clinical burdens, the path forward must involve deliberate strategies to broaden efficacy, ensure inclusivity, and uphold high ethical standards. Below are targeted recommendations distilled from the pilot’s core findings and the emergent themes across the earlier sections:

• Tailor Onboarding and Training Programs: The pilot showed that variations in user adaptability were partly tied to prior exposure to digital health solutions. To rectify this, SMILE’s developers should create multi-tiered onboarding modules that cater to distinct levels of technology proficiency. Simple, intuitive tutorials may suffice for those experienced with telehealth, while newly digitizing clinics could benefit from in-depth, step-by-step guides and structured mentoring. An emphasis on scenario-based training that underscores practical benefits—like stress reduction or improved care pathways—could foster quicker and more sustained adoption.
• Expand Partnerships with Underserved Communities: Given the pilot’s setting, many low-resource or rural contexts were not represented. Collaborating with healthcare organizations, NGOs, and government bodies in underserved regions is critical for proof-of-concept expansions. These partnerships should explore alternative connectivity solutions (e.g., low-bandwidth networks, caching mechanisms) to facilitate robust usage under challenging infrastructure conditions. Such expansions would also uncover any localized cultural nuances that might influence CBT acceptance or data privacy concerns, thereby encouraging further customization of SMILE’s modules and AI-driven features.
• Strengthen Algorithmic Oversight and Bias Audits: Ongoing algorithmic evaluations should be embedded in the platform’s lifecycle to identify any disparities in recommendation efficacy across diverse clinical populations. Employing fairness metrics, explainable AI frameworks and real-time feedback loops can help maintain system integrity. SMILE’s technical roadmap could integrate specialized subroutines that flag potential biases (e.g., systematically lower stress reduction for certain demographic groups) and automatically prompt re-training or data augmentation. Regularly published transparency reports documenting these audits would enhance trust among stakeholders and regulatory bodies, echoing a practice increasingly advocated for by AI ethics scholars.
• Advance Policy Engagement and Cross-Border Collaboration: To address the mosaic of regulatory and cultural norms, SMILE’s developers should actively engage with international consortia like the World Health Organization (WHO) and professional bodies in target regions. Such engagement would foster reciprocal learning, where the architecture could inform global standards for AI-driven mental health interventions, while local policymakers and experts can highlight region-specific compliance and interoperability requirements.
• Implement Ongoing Evaluation and Longitudinal Studies: While short-term gains in satisfaction and stress relief are evident, longitudinal research is paramount for confirming the enduring impact of SMILE interventions. Future studies might incorporate repeated measures (e.g., 6-month, 12-month follow-ups), delve into patient outcomes, and assess workforce retention. By elucidating how stress, satisfaction, or burnout trajectories evolve, the platform can refine its CBT modules, adapt scheduling prompts, and further optimize user experiences. This will also provide real-world evidence to show whether initial improvements hold or wane as novelty factors diminish.
• Broaden the Therapeutic Arsenal: Though CBT and NLP were well-received, expanding SMILE’s repertoire to encompass alternative psychotherapeutic methods—like mindfulness-based stress reduction or acceptance and commitment therapy, could widen its scope. Additionally, the pilot suggests that some clinicians might benefit from advanced functionalities, such as guided journaling tools, symptom diaries linked with wearable data, or AR-based interventions for acute stress relief. Ongoing dialogue with mental health professionals and AI specialists can steer future expansions that remain aligned with emerging clinical research.
• Cultivate User Communities and Peer Support Networks: A distinctive strength of SMILE has been its integrated peer support network, wherein clinicians can exchange coping strategies and experiences. Building robust online communities within the platform, supported by moderators or senior HCP “champions,” can help maintain consistent engagement and knowledge sharing. Over time, peer-based insight gathering (e.g., user-reported tips for mental health management) might serve as a valuable dataset for iterative platform enhancements, encouraging an organic community of practice that fosters continuous learning and emotional support.
• Address Technical Scalability and Performance Benchmarks: To reconcile the platform’s ambition with real-world constraints, SMILE must verify that its blockchain, federated learning modules, and AI sub-systems maintain high throughput and minimal latency. For instance, large-scale deployments with thousands of concurrent users will stress test any solution’s architecture. Ongoing performance benchmarking in increasingly complex environments—ranging from academic medical centers with robust infrastructures to small rural clinics with limited connectivity—will clarify the system’s scaling boundaries and prompt the adoption of microservices or containerization if necessary.
• Develop Transparent Governance and Clear Accountability: Finally, forging a clear accountability framework for both clinical decisions and data stewardship is essential. Clinicians should retain professional autonomy, with the platform serving as an informational ally rather than a prescriptive authority. Meanwhile, explicit governance mechanisms—covering data custody, blockchain ledger maintenance, and stakeholder agreements—need to be codified. Transparent governance ensures that any system errors, data breaches, or contested AI recommendations can be swiftly managed while preserving trust among clinicians, patients, and regulators.

The pilot’s positive results for SMILE lay a solid foundation for broader adoption, yet they also highlight the complexities of scaling an AI-driven, clinically oriented platform. By strategically addressing infrastructure disparities, ethical ramifications, algorithmic reliability, and user empowerment, SMILE can evolve from a promising pilot into a globally relevant tool for improving mental healthcare. The recommendations outlined above provide a multi-pronged blueprint for moving forward—balancing technical innovation, cultural sensitivity, and ethical rigor to ensure the platform’s sustained value in diverse healthcare landscapes.

## 6 Conclusion

The SMILE platform offers a pioneering, integrated response to the escalating challenges in mental health care. By merging advanced AI-driven analytics, blockchain-based identity and consent management, and clinically grounded modules for CBT and NLP, SMILE transcends conventional approaches in this domain. Its ability to streamline clinician workflows—reducing stress and administrative inefficiencies—while simultaneously fostering a more personalized, equitable, and ethically compliant system demonstrates the viability of technologically sophisticated yet user-oriented solutions. Central to SMILE’s success is its emphasis on collaboration. Through active engagement with clinicians, policymakers, technologists, and academic researchers from inception to deployment, the platform remains aligned with genuine clinical requirements and ethical imperatives. The iterative, multidisciplinary collaboration also bolsters user trust and ownership, facilitating smoother adoption in real-world settings. In parallel, SMILE’s rigorous attention to security and data privacy, including the use of blockchain-based consent ledgers and federated learning, supports robust, transparent governance that is critical for mental health applications where data sensitivity is paramount.

Moving forward, scalability and global adaptability are integral to the platform’s evolution. Features such as multilingual support, culturally tailored interventions, and flexible, resource-sensitive deployment models enable SMILE to transcend geographical and infrastructural barriers. These attributes position the platform well to address disparities in care across diverse regions and resource levels. Nonetheless, future work must address the persistent challenges of ensuring unbiased AI outputs, maintaining ethical oversight across heterogeneous regulatory landscapes, and bridging the digital divide in underserved communities. Continuous monitoring of algorithmic fairness, iterative user feedback loops, and thoughtful investments in training and infrastructural improvements will be vital for sustaining SMILE’s impact over the long term. In essence, SMILE stands as an illustrative example of how interdisciplinary innovation, grounded in real-world needs and rigorous ethical principles, can reshape the future of mental healthcare. Its modular architecture, spanning advanced AI analytics and educational resources, highlights the potential for integrated platforms to optimize both patient care and clinician well-being. By uniting technology and human-centered design, SMILE paves a credible pathway toward a more equitable, efficient, and resilient mental healthcare ecosystem, setting a benchmark for future solutions in this evolving field.

## Funding

Funding provided by ISCTE-IUL Business Research Unit. Funding reference ICMPD/2021/MPF-357-010

## Ethical statement

This study did not involve human participants or animal research that would require ethical approval. The data used for this research were either publicly available, anonymized, or generated for methodological validation purposes. All procedures complied with relevant regulations, including GDPR standards, to ensure data privacy and security.

## CRediT authorship contribution statement

**Pesqueira Antonio:** Writing – review & editing, Writing – original draft, Project administration, Methodology, Investigation, Conceptualization. **Sousa Maria:** Validation, Supervision. **Pereira Ruben:** Validation, Supervision, Methodology. **Schwendinger Mark:** Writing – review & editing, Validation, Formal analysis.

## Declaration of Competing Interest

The authors declare that they have no known competing financial interests or personal relationships that could have appeared to influence the work reported in this paper.

## Figures and Tables

**Table A.1:** Study Variables, Scales, and Operational Definitions

**Variable**	**Scale / Format**	**Operational Definition**	**Range**
Support Time	Minutes (continuous)	The total number of minutes an HCP spent seeking/providing clinical support (e.g., gathering information, interacting with colleagues) during a typical shift.	5–30 + minutes
Stress Score	Likert-type (1–10)	A self-reported measure of perceived stress levels in clinical practice, with 1 = “no stress” and 10 = “extreme stress.”	1–10 (integer)
Satisfaction Score	Likert-type (1–10)	Participant’s overall satisfaction with their work environment, digital resources, and mental health support systems, with 1 = “very dissatisfied” and 10 = “very satisfied.”	1–10 (integer)
Value Score	Likert-type (1–10)	Perceived utility of available resources/support systems, with 1 = “no value” and 10 = “high value.”	1–10 (integer)
Blockchain Consent Logging	Yes/No (binary usage)	Indicates whether an HCP or patient required a logged consent action via the permissioned blockchain for data access.	0–1 (per event)
Federated Learning Participation	Yes/No (binary usage)	Tracks whether a participant’s local data was used in the federated learning loop (e.g., for AI model training) without direct central data transfer.	0–1 (per participant)
CBT/NLP Module Usage	Count of sessions or time spent	Logs the number of times an HCP engaged with the CBT or NLP modules for stress relief, resilience building, or continuing education (CME) modules.	0–10 + sessions

**Table B.1:** Sample Focus Group Discussion Guide

**Topic / Question**	**Purpose**	**Example Probes**
Ease of Use and User Experience	Evaluate usability and interface satisfaction	“Which part of SMILE’s interface do you find most/least intuitive?”
Perceived Impact on Stress Levels	Gauge changes in workplace stress and emotional burden	“How has your stress level changed while using SMILE’s CBT features?”
Clinical Workflow Integration	Understand changes in daily tasks and information retrieval	“Are you finding it quicker to access guidelines or data now?”
Consent and Privacy	Assess understanding of blockchain-led data governance	“Do you feel more secure about patient data privacy with SMILE?”
Peer Support Module	Examine the utility of secure peer communication	“Has sharing experiences with colleagues helped reduce isolation?”
Overall Satisfaction and Future Improvements	Collect suggestions for refinements	“What enhancements would you recommend for a future SMILE release?”
